# The Importance of Gastropexy in Minimally Invasive Hiatal Hernia Repair

**DOI:** 10.3390/life16040598

**Published:** 2026-04-03

**Authors:** Razvan Calin Tiutiuca, Costel Bradea, Paulina Czidziak, Alina Ioana Nastase Puscasu, Cristian Dumitru Lupascu, Octav Ginghina, Mara Mardare, Valentin Bejan, Florina-Delia Andriesi-Rusu, Alin Mihai Vasilescu

**Affiliations:** 1 Faculty of Medicine, University of Medicine and Pharmacy “Gr. T. Popa” Iasi, 700115 Iasi, Romania; razvantiutiuca@gmail.com (R.C.T.); paula_cizzy@yahoo.com (P.C.); puscasu.alina92@gmail.com (A.I.N.P.); cristian.lupascu@umfiasi.ro (C.D.L.);; 2 “Saint Spiridon” Hospital Iasi, Independentei Str., No. 1, 700111 Iasi, Romania; 3 Faculty of Medicine, University of Medicine and Pharmacy “Carol Davila”, 020021 Bucharest, Romania

**Keywords:** hiatal hernia, gastropexy, gastroesophageal reflux

## Abstract

Hiatal hernia is a complex pathology, associated with gastroesophageal reflux disease, and for which management involves complex surgical treatment. Knowing the role of gastropexy in reducing postoperative recurrences, the current study aimed to highlight the intraoperative advantages and results of this surgical technique. Our study includes 29 patients aged between 34 and 84 years. Regarding the mechanism of occurrence, two thirds of the patients presented with mixed hiatal hernias (65.52%), 31.03% with sliding hiatal hernias, and 3.45% with paraesophageal hiatal hernias. The hernia size played a decisive role in the choice of surgical procedure, whereby for large hernias (with a diameter of over 7 cm), the Nissen procedure associated with gastropexy was preferred. For hernias with a diameter of less than 7 cm, the Nissen procedure associated with hernia orifice repair was performed. For hernias between 5 and 6 cm, gastropexy was also performed. Statistical analysis revealed a close correlation between the surgical procedure and the hernia size, with *p* = 0.005. Out of a total of 29 patients, 18 patients had gastropexy and 11 were without gastropexy. The association of gastropexy does not increase hospitalization costs, but in the long term, it has the advantage of reducing relapse.

## 1. Introduction

The management of hiatal hernias, particularly paraesophageal hernias, poses significant challenges, requiring precise and often complex surgical interventions. Hiatal hernias occur when a portion of the stomach protrudes through the esophageal hiatus into the thoracic cavity, commonly leading to gastroesophageal reflux disease (GERD), dysphagia, and, in severe cases, gastric volvulus or obstruction [1,2]. Among various surgical approaches, gastropexy has emerged as a crucial technique for enhancing surgical repair outcomes, particularly in large or complicated hernias [3,4].

The various types of hiatal hernias—Types I to IV—differ in their anatomical characteristics and associated complications. Types III and IV are more prone to severe complications, which often require surgical intervention to prevent potentially life-threatening scenarios [2,5]. The role of gastropexy in preventing recurrence and facilitating a more favorable recovery trajectory has garnered attention in recent research, emphasizing its importance as part of the surgical approach to managing hiatal hernias [3,6].

Hiatal hernias are characterized by the abnormal displacement of stomach contents into the thoracic cavity, predominantly due to a weakening of the diaphragm and surrounding musculature. This condition is often closely associated with GERD, which can manifest heartburn, regurgitation, and dysphagia [1,7]. In symptomatic cases, especially in which complications arise, surgical intervention is often deemed necessary. Laparoscopic hiatal hernia repair is currently recognized as the gold standard due to its minimally invasive nature, which typically results in reduced postoperative pain and quicker recovery times compared to traditional open surgeries [8,9].

The standard procedure for laparoscopic repair generally involves closing the hiatal defect, often accompanied by an anti-reflux procedure such as Nissen fundoplication [10]. However, the effectiveness of these repairs can be significantly compromised if the stomach is not adequately secured postoperatively, leading to recurrence. This realization has highlighted the relevance of gastropexy, which involves surgically securing the stomach to the abdominal wall, thus maintaining its proper anatomical position and mitigating the risk of herniation.

Gastropexy presents a particular advantage for elderly patients or those with significant comorbidities who may present greater risks during extensive surgical maneuvers [6]. By securing the stomach post-repair, gastropexy can contribute to reduced complications and enhanced recovery profiles for these patients [3,4]. Evidence increasingly indicates that employing anterior gastropexy significantly improves outcomes for high-risk patients, particularly in preventing the recurrence of large hiatal hernias [4].

## 2. Material and Methods

A retrospective observational study was conducted at a tertiary referral center for upper gastrointestinal surgery. All consecutive patients who underwent elective minimally invasive hiatal hernia repair between January 2019 and December 2023 were evaluated. The study was designed to assess the role of gastropexy as an adjunctive procedure in laparoscopic hiatal hernia repair, with particular emphasis on early recurrence and postoperative symptom persistence.

Patient Selection.

Inclusion criteria:

- Age ≥ 18 years.
- Symptomatic hiatal hernia confirmed by upper gastrointestinal endoscopy and/or computed tomography.
- Elective laparoscopic hiatal hernia repair.
- Complete perioperative and follow-up data.

Exclusion criteria:

- Previous esophageal or gastric surgery.
- Emergency procedures.
- Concomitant bariatric surgery.
- Follow-up shorter than six months.

Data Collection.

Clinical data were extracted from electronic medical records and operative reports. The following variables were analyzed:

- Demographic data (age, sex).
- Body mass index (BMI).
- Comorbidities (obesity, diabetes mellitus, cardiovascular, and pulmonary disease).
- Duration and type of symptoms (heartburn, regurgitation, dysphagia, chest pain).
- Type of hiatal hernia (sliding, mixed, paraesophageal).
- Hiatal defect size measured intraoperatively (maximum transverse diameter).
- Surgical technique employed.
- Operative time.
- Length of postoperative hospital stay.
- Postoperative complications.
- Early hernia recurrence.

Surgical Technique.

All procedures were performed laparoscopically by experienced upper gastrointestinal surgeons. After reduction of the hernia sac and mobilization of the distal esophagus, posterior crural closure was performed using non-absorbable sutures. A 360° Nissen fundoplication was completed in all cases.

Gastropexy was selectively performed based on intraoperative assessments, particularly in patients presenting with:

- Hiatal defects ≥ 5 cm.
- Significant intrathoracic stomach migration.
- Esophageal shortening or increased tension at the hiatal repair.

Anterior gastropexy consisted of fixation of the gastric fundus to the anterior abdominal wall using non-absorbable sutures, aiming to limit postoperative axial migration of the stomach and reduce tension on the hiatal closure.

Outcomes.

The primary outcome was early hiatal hernia recurrence, defined as radiological or symptomatic evidence of herniation within six months postoperatively.

Secondary outcomes included:

- Persistence of postoperative dysphagia.
- Operative time.
- Length of hospital stays.
- Perioperative complications.

Postoperative follow-up was performed for all patients, both symptomatic and asymptomatic. The surgical team followed the patients postoperatively using upper digestive endoscopy, barium swallow, and computer tomography examination. All patients returned for follow-up at 6 months postoperatively. Follow-up at 1 or 2 years postoperatively is under evaluation.

Statistical Analysis.

Statistical analysis was performed using SPSS software (IBM SPSS Statistics 20). Continuous variables were expressed as mean ± standard deviation or median (range), depending on distribution. Comparisons between groups were performed using Student’s *t*-test or Mann–Whitney U test for continuous variables and chi-square or Fisher’s exact test for categorical variables.

Receiver operating characteristic (ROC) curve analysis was used to assess the predictive value of hiatal hernia size for persistent dysphagia. A *p*-value < 0.05 was considered statistically significant.

## 3. Results

Patient Demographics.

**Figure 1:**
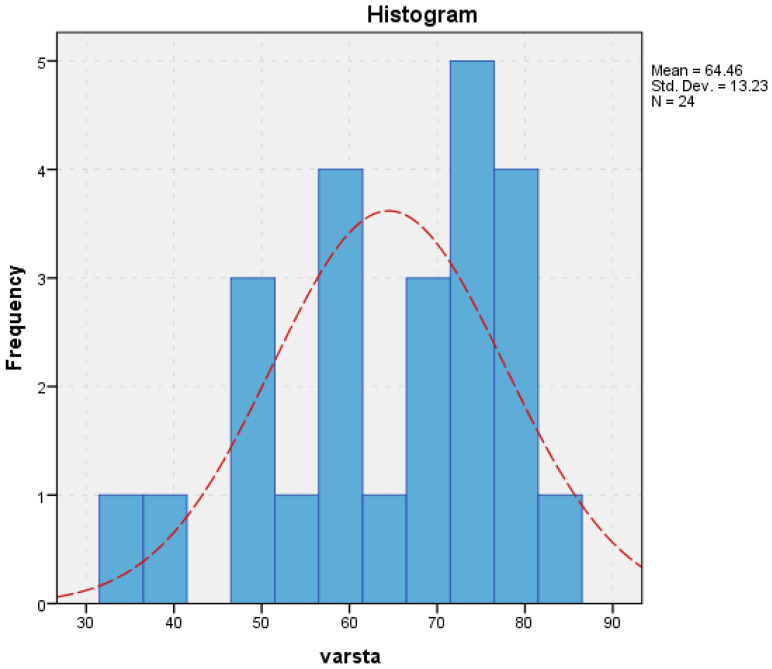
Age histogram (varsta = age, N = number of patients).

A total of 29 patients were included in the study, with ages ranging from 34 to 84 years and a median age of 68 years. Female patients predominated (75.9%, *n* = 22), resulting in a female-to-male ratio of approximately 3:1. Half of the study population consisted of elderly patients (>68 years), reflecting the known age-related prevalence of hiatal hernia (Figure 1).

Gender-based analysis demonstrated a lower mean age among female patients compared to males; however, no statistically significant differences were observed regarding symptom duration or timing of surgical intervention between genders (Table 1).

This difference cannot be attributed to women presenting to the doctor earlier, but to the fact that this pathology occurs in men at an older age. This hypothesis is supported by the data in Table 2, which highlights the fact that there are no differences between women and men in terms of the duration between the onset of symptoms and the time of surgery.

A majority of the patients within this cohort exhibited a prolonged history of gastroesophageal reflux disease (GERD), with symptom durations ranging from 5 to 12 years, reflecting significant chronicity. The prevalent comorbidities included obesity (32% prevalence), diabetes (21%), cardiovascular diseases (28%), and pulmonary comorbidities (18%). These comorbid factors are essential considerations, as they may profoundly impact surgical outcomes, as Almutairi et al. and Arcerito et al. noted [7,11].

**Figure 2:**
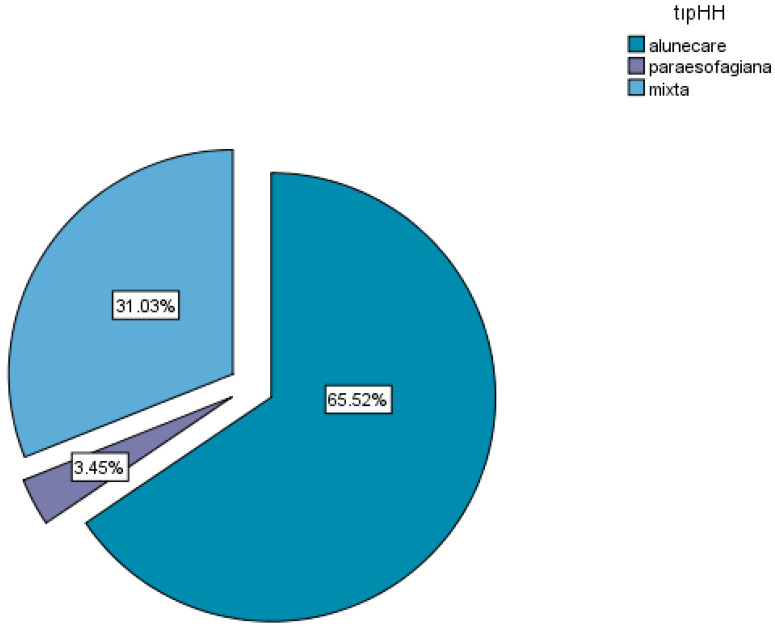
Types of hiatal hernias. tipHH = hiatal hernia type; alunecare = sliding hernia; paraesofagiana = paraesophageal hernia type.

Regarding the mechanism of occurrence, two thirds of the patients presented with mixed hiatal hernias (65.52%), 31.03% with sliding hiatal hernias, and 3.45% with paraesophageal hiatal hernias (Figure 2).

Hiatal hernia represents a widespread clinical condition that frequently requires surgical intervention. The size of the hiatal defect can carry substantial clinical implications, influencing not just patient characteristics but also the severity of symptoms, the complexity of surgical procedures, and the outcomes post-surgery. This study aims to rigorously explore the correlations between the size of the hiatal defect and a variety of factors, including patient demographic variables, comprehensive histories of gastroesophageal reflux disease (GERD), durations of symptoms, surgical time, length of hospitalization, and the surgical techniques used.

**Figure 3:**
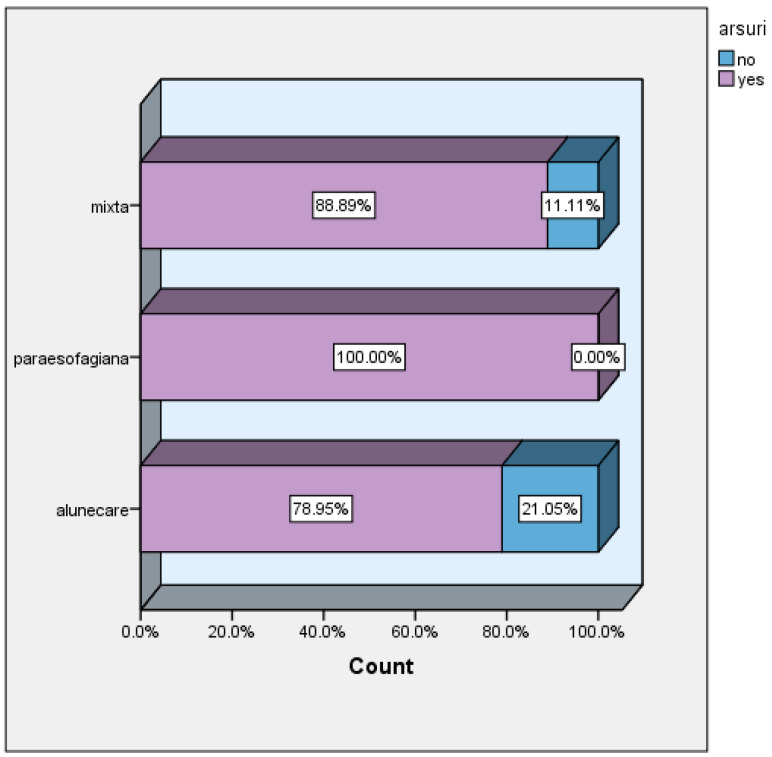
Heartburn versus hernia type, arsuri = heartburn; alunecare = sliding hernia; paraesofagiana = paraesophageal hernia.

Heartburn was present in all patients with paraesophageal hiatal hernia, in 88.89% of patients with mixed hiatal hernia, and in 78.95 of patients with sliding hernia, with this being the predominant symptom (Figure 3).

**Figure 4:**
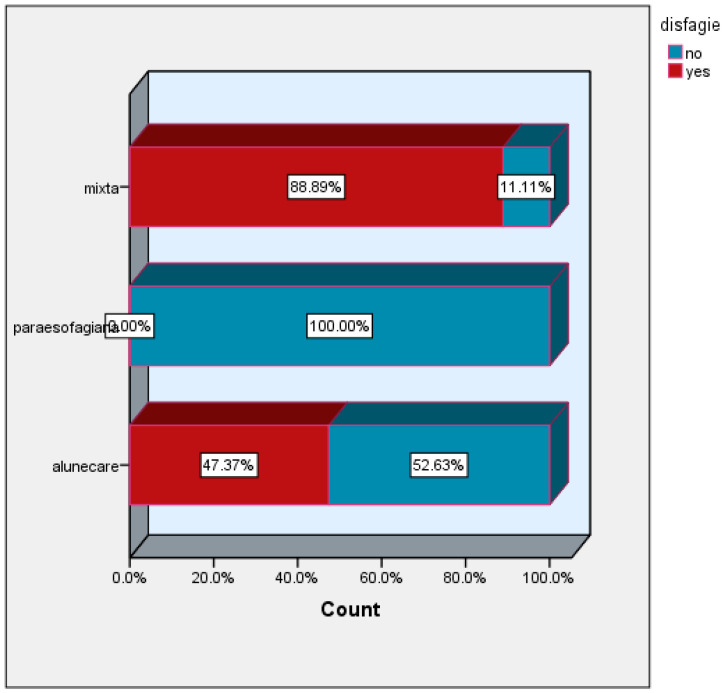
Dysphagia versus hernia type, disfagie = disphagia; alunecare = sliding hernia; paraesofagiana = paraesophageal hernia.

The incidence of dysphagia was 88.89% in the group of patients with mixed hiatal hernias and 47.37% in the group of patients with sliding hiatal hernias. This symptom was absent in the group of patients with paraesophageal hiatal hernias (Figure 4).

**Figure 5:**
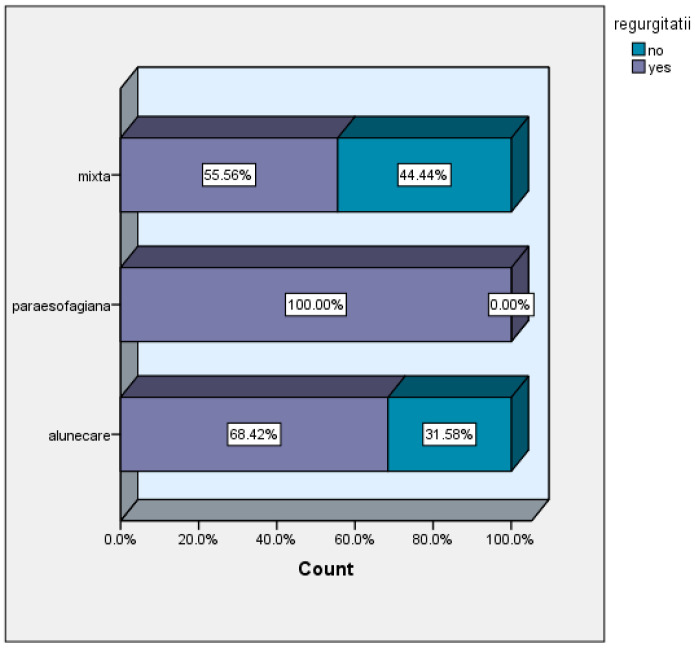
Regurgitation versus hernia type, regurgitatii = regurgication; alunecare = sliding hernia; paraesofagiana = paraesophageal hernia.

The incidence of regurgitation was approximately equivalent in the group of patients with mixed and sliding hiatal hernias (55.56% and 68.42%, respectively), unlike in the group of patients with paraesophageal hernia, where all presented this symptom (Figure 5).

**Figure 6:**
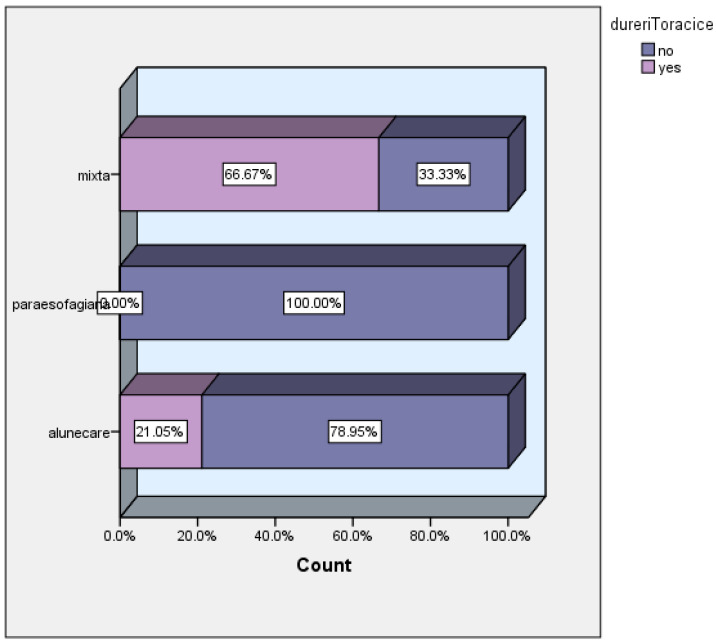
Chest pain versus hernia type, dureritoracice = chest pain; alunecare = sliding hernia; paraesofagiana = paraesophageal hernia.

Two-thirds of patients with mixed hiatal hernias experienced chest pain, which was three times more than in the group of patients with sliding hernias (Figure 6).

Preoperative treatment versus types of hiatal hernia.

**Figure 7:**
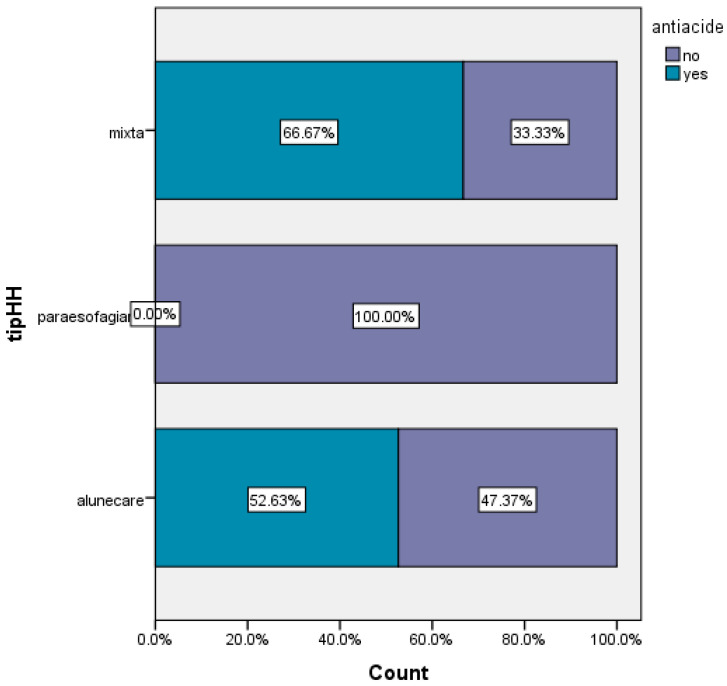
Preoperative treatment versus hernia type, antiacide = antacid treatment; alunecare = sliding hernia; paraesofagiana = paraesophageal hernia.

Two-thirds of patients with mixed hiatal hernias and 52.63% of patients with sliding hiatal hernias, required treatment with antacids (Figure 7).

**Figure 8:**
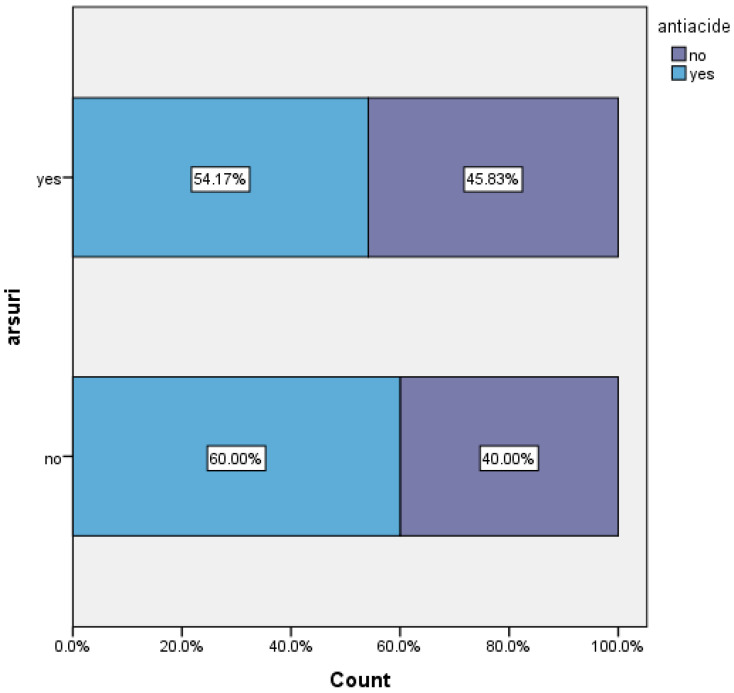
Antacid versus persistence of heartburn, arsuri = heartburn; antiacide = antiacid treatment.

60% of patients who did not experience heartburn received antacid treatment, which may suggest that for these patients, the treatment was effective in controlling symptoms. Among patients with persistent heartburn, only 54.17% continued antacid therapy (Figure 8).

Hernia size versus presence of symptoms.

No statistical correlations were found between hernia size and the presence of retrosternal heartburn, regurgitation, or chest pain.

Statistical analysis identified a strong statistical correlation between hiatal hernia size and the persistence of dysphagia (*p* = 0.004) (Table 3).

**Figure 9:**
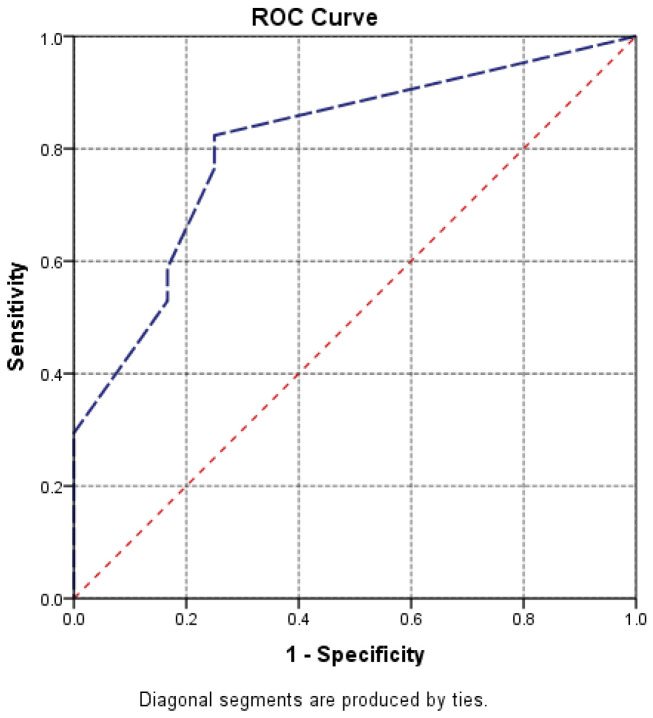
Dysphagia vs. hernia size.

Also, the analysis of the ROC curve and AUC = 0.809 indicates the high predictive power of hernia size on the persistence of dysphagia and lack of response to treatment (Figure 9, Table 4). It follows that, as the hernia increases in size, we expect dysphagia to persist even if conservative treatment is administered, and for the response to this drug treatment to be minimal.

Hernia size and choice of surgical intervention.

Statistical analysis revealed a close correlation between the surgical procedure and the hernia size, with *p* = 0.005.

**Figure 10:**
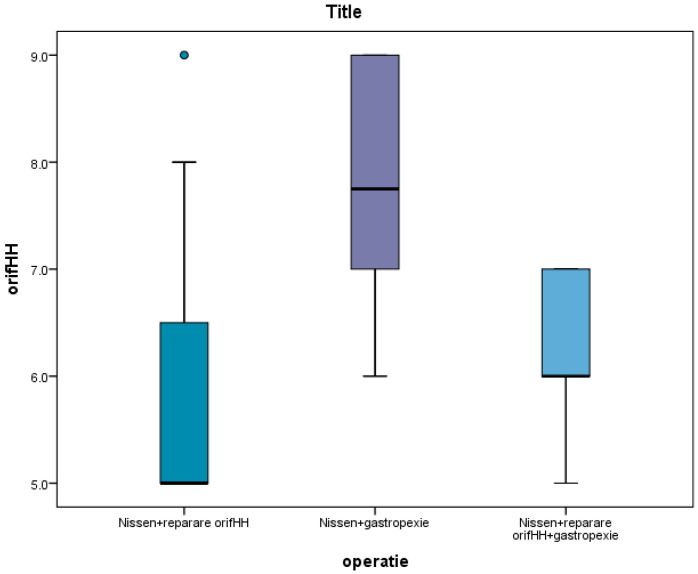
Hernia size versus surgical procedure. orifHH = hiatal hernia orifice; Nissen + reparare orifHH = Nissen procedure associated with hiatal hernia orifice repair; Nissen + gastropexie = Nissen procedure associated with gastropexy; Nissen + reparare orifHH + gastropexie = Nissen procedure associated with gastropexy and hiatal hernia orifice repair.

As can be seen in the image below, hernia size played a decisive role in the choice of surgical procedure; for large hernias (with a diameter of over 7 cm), the Nissen procedure associated with gastropexy was preferred (Figure 10).

For hernias with a diameter of less than 7 cm, the Nissen procedure associated with hernia orifice repair was performed. For hernias between 5 and 6 cm, gastropexy was also performed (Figure 10).

**Figure 11:**
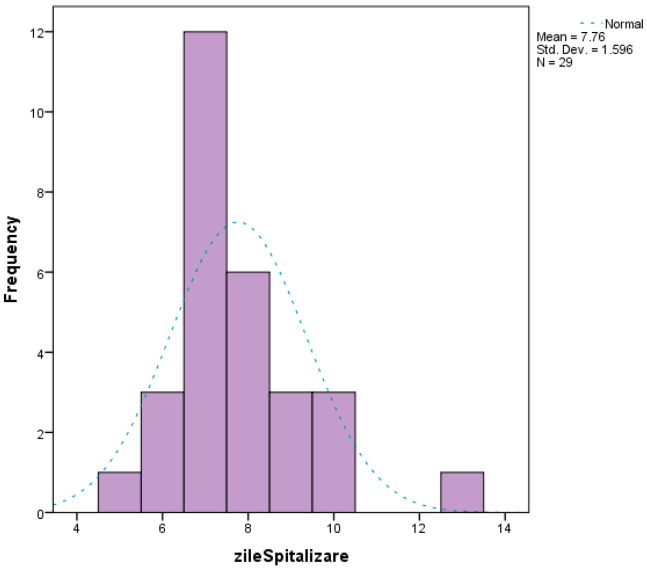
Surgical procedure vs. hospitalization. Zile spitalizare = number of hospitalizations days.

The choice of surgical procedure did not influence either the duration of the operation or the number of days of hospitalization, with no statistical associations between these variables. The duration of hospitalization ranged from 5 to 13 days, with a median of 7 days (Figure 11).

In these conditions, it is preferable to use gastropexy considering that there is no significant increase in intraoperative time, nor in the number of days of hospitalization. The association of gastropexy does not increase hospitalization costs, but, in the long term, has the advantage of reducing relapses.

The influence of obesity in study group.

**Figure 12:**
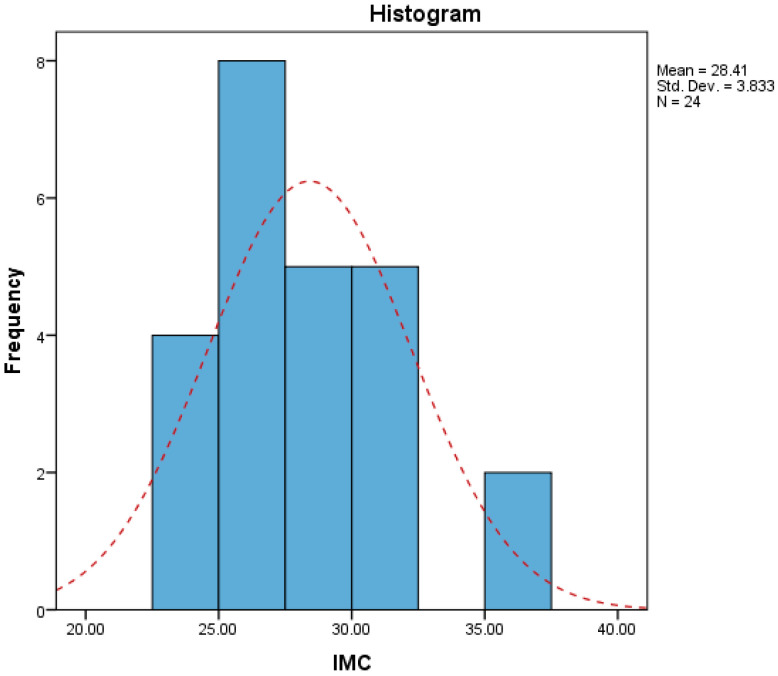
Distribution of obesity in the study group. IMC = body mass index.

The patients’ BMI ranged between 22.96 and 36.79, with a median of 27.49 (Figure 12).

No statistically significant correlations were identified between BMI and hiatal hernia size.

**Figure 13:**
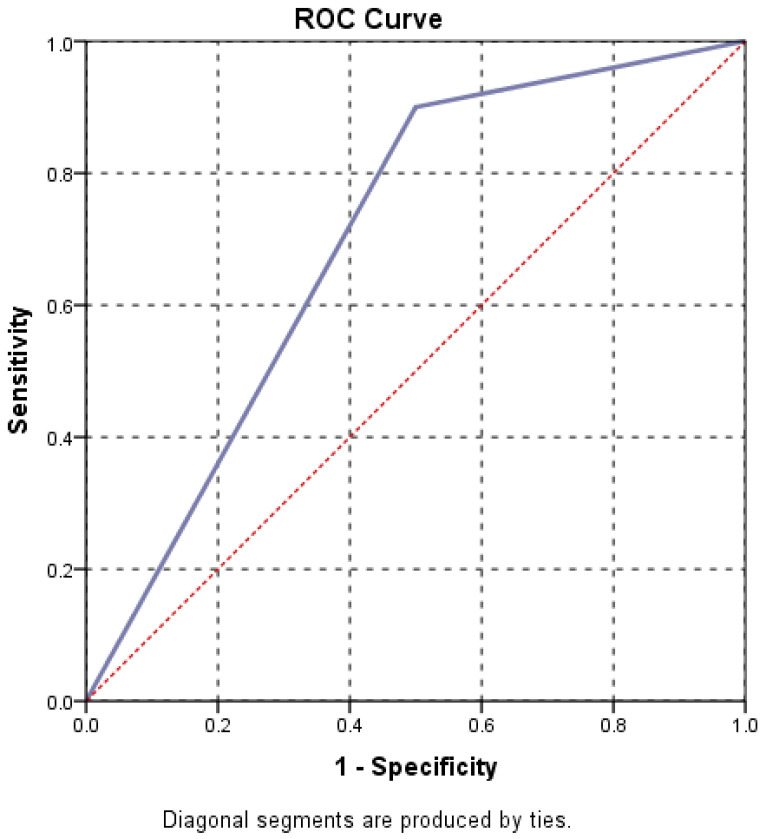
Obesity vs. retrosternal heartburn.

ROC curve analysis revealed that a BMI ≥ 25 has a high predictive value on the occurrence and persistence of retrosternal heartburn, with an AUC = 0.700 (Figure 13). Of all the symptoms present in patients, obesity was only correlated with the presence of retrosternal pain. The results show that the presence of obesity and its maintenance will determine the persistence and recurrence of retrosternal pain.

BMI does not influence and does not show statistical correlations with other symptoms.

**Figure 14:**
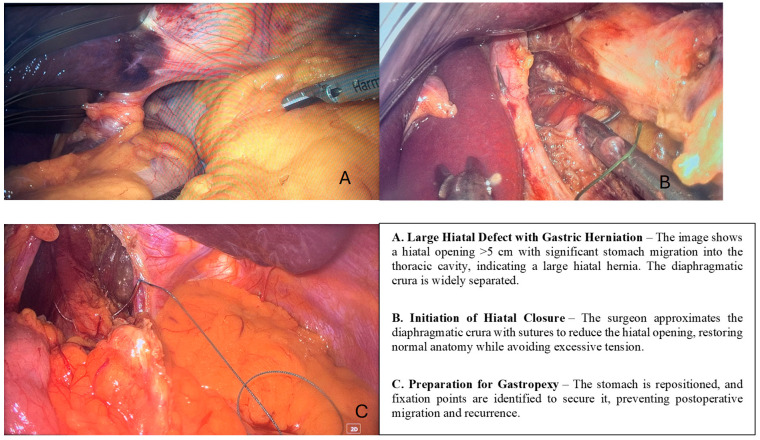
Intraoperative aspect.

Intraoperative section and surgical technique details (Figure 14):

Surgical Techniques and Outcomes: All patients included in this robust study underwent laparoscopic hiatal hernia repair, with each individual receiving the widely recognized Nissen fundoplication procedure, which remains a standard technique for effectively addressing hiatal hernias. Within this carefully selected cohort, selective gastropexy was performed in approximately 62% of cases, specifically targeting patients with additional complications such as gastric volvulus, considerable intrathoracic stomach migration, or significant esophageal shortening.

## 4. Discussions

**The Role of Gastropexy in Recurrence Prevention**.

The treatment of hiatal hernias was and remains a very controversial topic. There is important heterogeneity in studies and in the techniques and technologies employed. Although the first surgery was described in 1919 by Soresi [12], each new approach has been viewed with extreme enthusiasm, only for most to not withstand the scrutiny of time [13].

Traditional surgical methods have raised concerns regarding morbidity rates, with laparoscopic techniques demonstrating significantly lower complication rates compared to open repair strategies [14]. The lower complication rate comes in with a very high recurrence rate of up to 50% [15]. A low percentage, 0.01 to 7%, of these patients require revisional surgery, but they exhibit a worse quality of life and increased symptoms of heartburn, early satiety, gas bloat, and dysphagia [16,17].

There are still a lot of controversies regarding the adequate surgical treatment of hiatal hernial, especially with regard to which procedure has the lowest recurrence rate as compared to crural sutures alone. Multiple concomitant procedures have been described, all very enthusiastically advocated for at first, but without long term assessment. One such instance is the example of using tension-free polyurethane mesh in addition to the crural suture [18]. A randomized clinical study aimed to assess long term (13 years) anatomical and functional outcomes in 103 patients, from the original 158 enrolled in the double-blind study. Radiologically, there was no significant difference between recurrence rates, with 38% for mesh and 31% for suture alone. Worth mentioning are the mechanical complications. There were also significantly higher dysphagia scores for solids in the mesh group. Other complications are mesh erosion, dense fibrosis leading to dysphagia, chronic pain, or gastroparesis [19].

The next logical approach is the use of biosynthetic meshes alongside crural suture. Available studies have shown significant improvement in quality of life scores, despite extremely variable recurrence rates varying from 0.9 to 25%. This can be attributed to the heterogeneity of the studies [20].

Another therapeutical approach which is currently being used in the treatment of gastroesophageal reflux is the concomitant fundoplication. There is an absence of strong data to support the long-term effects, although it is under the assumption that it can prevent or treat the reflux while fixing the stomach below the diaphragm and thus preventing recurrence [21]. Available data shows that fundoplication can prevent postoperative reflux and subsequent esophagitis, especially in patients for which there is an incompetence of the lower esophageal sphincter due to extensive hiatal dissection during hernia repair. Another controversial topic is the type of fundoplication. Most studies did a total fundoplication (Nissen or Nissen-Rosetti), and the latest guidelines recommend that partial fundoplication (Toupet of Dor) should be reserved for cases with significantly impaired esophageal mobility [22].

Gastropexy without the repair of the hiatal hernia was first described in 1955 by Boerema and Germs. The hernia sac was not or was minimally dissected, the stomach was reintroduced into the peritoneal cavity, and the esophagus and stomach were put under tension through gastropexy of the lesser sac to the right of the laparotomy incision with multiple sutures [23]. This method, although first showing promising results, has been abandoned for years because of the high recurrence rates; this can be attributed to the lack of vital techniques such as crural suture or the reduction of the hernia sac [24].

Different types of gastropexy have been described, such as posterior gastropexy (in which the stomach is sutured to the diaphragmatic crura), T-fastner percutaneous gastropexy (a T-fastener is placed into the gastric lumen and secured externally; once the external sutures are cut, the T-fastener is able to migrate through the gastrointestinal tract and be expelled naturally), or using a percutaneous endoscopic gastrostomy tube as an anterior gastropexy in emergency settings has been proposed [24,25,26].

One study aimed at assessing the relevance and late results of laparoscopic hernia repair without prosthetic reinforcement concluded that a factor significantly linked with recurrence was the absence of gastropexy (50% as compared to 10.8% in the group with gastropexy) [27]. These results have been confirmed by multiple other studies in smaller and non-comparative studies [28,29,30,31,32,33]. The anatomical hypothesis is that anchoring the stomach to the anterior wall will prevent reherniation and avoid organoaxial rotation with the risk of strangulation [34,35,36].

To add more to this already controversial topic, a recent study has shown no advantage of fundoplication as compared to gastropexy alone. The only difference was the median operation time (108 min for fundoplication compared to 59 min), but with comparable results regarding perioperative and postoperative events, reflux control, and recurrence [36].

Anterior gastropexy is very appealing due to the fact that it is easily performed laparoscopically, with a very low complication rate and easily reproducible [8]. This is reflected in our study, in which there were no complications reported, and there was also no difference in hospitalization time.

Incorporating gastropexy into standard hiatal hernia repair has been demonstrated to provide several significant advantages, such as the optimization of surgical outcomes in high-risk patients [13,37]. Our analysis reveals that larger hiatal defects correlate with extended surgical times (+0.30 correlation) and lengthier hospital stays (+0.40 correlation), suggesting a more intricate operative course. Incorporating gastropexy can act as a stabilizing adjunct in situations where conventional fundoplication alone may not suffice to deliver long-term support.

There are some limitations to the present study which need to be addressed. This is a single-center retrospective study spanning 4 years, and although the study cohort is robust, the results could be improved through a larger cohort. Another limitation is the fact that the surgical approach was tailored by the preoperative or intraoperative findings, especially by the size of the defect. All defects which were over 5 cm or any type of complication of the hernia included gastropexy as part of the surgical treatment. Considering the lack of consensus in the literature and the demographics of our cohort, gastropexy alone would not have been warranted. Therefore, the study lacks a control group with gastropexy alone. This would also have been outside the scope of the present study. The aim of the study was to enforce the use of gastropexy as a part of the surgical management of patients with hiatal hernias, as a safe, easy-to-perform procedure, while associating better outcomes. Thus, the low recurrence rates described in this paper do not solely reflect the recurrence rates of gastropexy alone; rather, a low recurrence when combined with an anti-reflux procedure.

Although there is an increasing body of evidence advocating gastropexy, further studies are necessary to clarify its role in surgical practice. Key areas for future research should include:

1. Comparative studies evaluating long-term recurrence rates with versus without gastropexy in cases involving large hiatal hernias. While existing retrospective data suggest potential benefits, randomized controlled trials would generate more definitive conclusions.
2. Investigation into alternative fixation techniques, such as mesh-reinforced repairs, alongside gastropexy, to discover the most effective treatment strategy for large or recurrent hiatal hernias.
3. Exploration of the role of gastropexy in individuals exhibiting altered esophageal motility, including those diagnosed with achalasia or ineffective peristalsis, to determine if additional gastric fixation contributes to improved symptom management.

In light of our findings and the current body of literature, we propose the following recommendations regarding the application of gastropexy in laparoscopic hiatal hernia repair:

1. Surgeons should contemplate the use of gastropexy for patients with extensive hiatal defects (greater than 5 cm), particularly in cases involving mixed-type hernias or increased intra-abdominal pressure.
2. Gastropexy should be embraced as a preventive strategy to diminish the chances of recurrence, particularly when high tension at the hiatal level exists, or stability following fundoplication is uncertain.
3. Timely surgical intervention in patients presenting with large hiatuses may help mitigate prolonged symptomology and reduce the risk of severe complications such as gastric volvulus.
4. A tailored approach should be adopted, with preoperative evaluations assessing the severity of GERD, esophageal motility, and anatomical risk factors guiding the decision to incorporate gastropexy.

The average duration of postoperative hospital stays among the patient cohort was approximately 8.2 days, a statistic that was notably influenced by many variables, including each patient’s preoperative health status, comorbid conditions, and the complexity of the surgical procedure performed. Importantly, throughout the study, no major complications—such as the formation of fistulas or significant postoperative bleeding—were documented within the studied population. However, minor complications emerged, with transient dysphagia occurring in 15% of patients and gas bloat syndrome being observed in 10% of patients.

The integration of gastropexy into laparoscopic hiatal hernia repair marks a significant advancement in the surgical management of complex hiatal defects. Although its routine utilization remains a point of contention, growing evidence supports its efficacy in lowering recurrence rates, enhancing symptom management, and improving surgical outcomes. As the field progresses, continuous research and extended follow-up studies will be vital in refining its indications and establishing standardized guidelines for its application.

## 5. Conclusions

Gastropexy represents a valuable adjunct in laparoscopic hiatal hernia repair, particularly in patients with large or mixed-type hernias. Hiatal hernia size plays a central role in symptom persistence and surgical decision-making, and larger defects may benefit from additional gastric fixation to enhance anatomical stability.

The selective use of gastropexy does not increase perioperative morbidity and may reduce early recurrence by limiting postoperative gastric migration and tension at the hiatal repair. These findings support a tailored surgical approach based on anatomical risk factors rather than the routine application of a single technique.

Prospective, randomized studies with longer follow-ups are required to further define the role of gastropexy and establish standardized indications for its use in minimally invasive hiatal hernia repair.

## Figures and Tables

**Table 1:** Age versus gender.

Gender	Mean	N	Std. Dev.	Median	Min	Max
F	62.79	22	11.98	64	34	79
M	70.80	7	17.22	77	41	84
Total	64.46	29	13.23	68	34	84

F = female; M = male; N = number of patients.

**Table 2:** Gender distribution versus duration between symptom onset and surgery.

Gender	Mean (Onset of Symptoms)	N	Std. Dev.	Median	Min	Max
F	9.55	22	3.113	10	4	15
M	11.50	7	4.087	10	8	18
Total	9.96	29	3.361	10	4	18

F = female; M = male; N = number of patients.

**Table 3:** Hernia size versus dysphagia.

Size of Hiatal Hernia Versus Dysphagia							
Dysphagia	Mean	N	Std. Dev.	Min	Max	Median	*p* Value
No	5.417	12	0.7930	5	7	5	0.004
Yes	6.853	17	1.4226	5	9	7	
Total	6.259	29	1.3863	5	9	6	
F = 9.970							

**Table 4:** Dysphagia vs. hernia size.

Area Under the Curve				
Test Result Variable(s): orifHH				
Area	Std. Error ^a^	Asymptotic Sig. ^b^	Asymptotic 95% Confidence Interval	
			Lower Bound	Upper Bound
0.809	0.082	0.005	0.647	0.970

The test result variable(s): orifHH has at least one tie between the positive actual state group and the negative actual state group. Statistics may be biased. ^a^. Under the nonparametric assumption. ^b^. Null hypothesis: true area = 0.5.

## Data Availability

The original contributions presented in this study are included in the article. Further inquiries can be directed to the corresponding authors.
